# Methylation of *DACT2* Promotes Papillary Thyroid Cancer Metastasis by Activating Wnt Signaling

**DOI:** 10.1371/journal.pone.0112336

**Published:** 2014-11-06

**Authors:** Zhiyan Zhao, James G. Herman, Malcolm V. Brock, Jindong Sheng, Meiying Zhang, Baoguo Liu, Mingzhou Guo

**Affiliations:** 1 The Department of Head & Neck Surgery, Peking University Cancer Hospital and Institute, #52 Fucheng Road, Beijing 100036, China; 2 The Sidney Kimmel Comprehensive Cancer Center at Johns Hopkins, The Bunting-Blaustein Cancer Research Building, Room 543, 1650 Orleans Street, Baltimore, Maryland 21231, United States of America; 3 The Department of Gastroenterology & Hepatology, Chinese PLA General Hospital, #28 Fuxing Road, Beijing 100853, China; 4 The Medical College of Nan Kai University, #94 Weijin Road, Tianjin 300071, China; University of Navarra, Spain

## Abstract

Thyroid cancer is the most common endocrine malignant disease and the incidence is increasing. *DACT2* was found frequently methylated in human lung cancer and hepatocellular carcinoma. To explore the epigenetic change and the role of *DACT2* in thyroid cancer, 7 thyroid cancer cell lines, 10 cases of non-cancerous thyroid tissue samples and 99 cases of primary thyroid cancer samples were involved in this study. DACT2 was expressed and unmethylated in K1, SW579, FTC-133, TT, W3 and 8505C cell lines. Loss of expression and complete methylation was found in TPC-1 cells. Restoration of DACT2 expression was induced by 5-aza-2′deoxycytidine treatment. It demonstrates that the expression of *DACT2* was regulated by promoter region methylation. In human primary papillary thyroid cancer, 64.6% (64/99) was methylated and methylation of DACT2 was related to lymph node metastasis (p<0.01). Re-expression of *DACT2* suppresses cell proliferation, invasion and migration in TPC-1 cells. The activity of TCF/LEF was inhibited by DACT2 in wild-type or mutant β-catenin cells. The activity of TCF/LEF was increased by co-transfecting DACT2 and Dvl2 in wild-type or mutant β-catenin cells. Overexpression of wild-type β-catenin promotes cell migration and invasion in DACT2 stably expressed cells. The expression of β-catenin, c-myc, cyclinD1 and MMP-9 were decreased and the level of phosphorylated β-catenin (p-β-catenin) was increased after restoration of DACT2 expression in TPC-1 cells. The expression of β-catenin, c-myc, cyclinD1 and MMP-9 were increased and the level of p-β-catenin was reduced after knockdown of DACT2 in W3 and SW579 cells. These results suggest that DACT2 suppresses human papillary thyroid cancer growth and metastasis by inhibiting Wnt signaling. In conclusion, *DACT2* is frequently methylated in papillary thyroid cancer. DACT2 expression was regulated by promoter region methylation. *DACT2* suppresses papillary thyroid cancer proliferation and metastasis by inhibiting Wnt signaling.

## Introduction

Thyroid cancer is the most common endocrine malignancy, and its incidence is increasing very fast globally [1]. Follicular epithelial cell-derived thyroid cancer was categorized into three histological types, including papillary thyroid cancer (PTC, 80%), follicular thyroid cancer (FTC, 15%), and anaplastic thyroid cancer (ATC, 2–5%). While the medullary thyroid carcinoma (MTC), which is developed from parafollicular C cells, is very rare [2]–[4]. Although the prognosis of thyroid carcinoma is much better, it is still very hard to select therapeutic method. The strategy of PTC treatment mainly includes surgical resection, adjunctive radioiodine ablation and thyrotropin suppression. The extent of thyroidectomy and lymphadenectomy remains controversial [5]. Epigenetic changes may serve as detective, prognostic and therapeutic marker in thyroid cancer.

Dapper, a Dishevelled-associated antagonist of β-catenin (DACT), was isolated by a screen for proteins interacting with Dishevelled, a key factor in the Wnt signaling. Dapper and Dishevelled were co-localized intracellularly and formed a complex with Axin, GSK3 and β-catenin [6]. Human DACT2 was identified by Katoh et al. and located on human chromosome 6q27 [7]. Waxman JS and Li Xiao et al. found that DACT2 promotes Wnt signaling during development in zebrafish and mouse teeth [8], [9]. Our previous studies found that DACT2 is a Wnt/β-catenin signaling inhibitor in lung and hepatocellular carcinoma [10], [11]. In this study, we analyzed the epigenetic change and the function of DACT2 in papillary thyroid cancer.

## Materials and Methods

### Ethics Statement

The study was performed in accordance with the guidelines of the 1975 Declaration of Helsinki and consistent with local regulatory requirements and good clinical practice guidelines. All samples were collected under the approved guidelines of Beijing Cancer Hospital’s institutional review board. All thyroid cancer cell lines were described previously [12]–[14]. The experimental methods were approved by the Ethics Committee of the Chinese PLA General Hospital (Permit Number: 20090701-015 and 20140423-001).

### Primary human papillary thyroid cancer samples and cell lines

A total of 99 cases of primary papillary thyroid cancer and 10 cases of normal thyroid tissue were collected as fresh frozen tissue from Beijing Cancer Hospital. All samples were collected under the approved guidelines of Beijing Cancer Hospital’s institutional review board. 7 thyroid cancer cell lines (K1, TPC-1, SW579, FTC-133, TT, W3 and 8505C) were included in this study. All thyroid cancer cell lines were previously established from primary thyroid cancer. K1, TPC-1, SW579, FTC-133 and TT cell lines were maintained in 90% RPMI 1640 (Invitrogen, Carlsbad, CA, USA) supplementing with 10% fetal bovine serum (FBS) at 37°C with 5% CO2. W3 and 8505C cell lines were maintained in 90% DMEM (Invitrogen, Carlsbad, CA, USA) supplementing with 10% fetal bovine serum at 37°C with 5% CO2. Cells were passaged 1∶3 once 80% confluence was reached on a 75 cm2 culture flask (NEST Biotechnology, shanghai, China). All thyroid cancer cell lines used in this study were reported previously [12]–[14].

### 5-aza-2′-deoxycytidine treatments

Thyroid cancer cell lines were split to low density 12 hours before treatment. Cells were treated with 5-aza-2′-deoxycytidine (5-Aza) (Sigma, St Louis, MO, USA) at a concentration of 2 µM in the growth medium, which was exchanged every 24 h for a total 96 hours treatment. At the end of treatment course, RNA was extracted.

### RNA isolation and regular PCR

Total RNA was isolated using Trizol reagent (Life Technologies, Gaithersburg, MD, USA). Agarose gel electrophoresis and spectrophotometric analysis were used to evaluate RNA quality and quantity. First strand cDNA was synthesized according to manufacturer’s instruction. A total of 5 µg total RNA was used to synthesize first strand cDNA using the Superscript III-reverse transcriptase kit (Invitrogen, Carlsbad, CA, USA). The reaction mixture was then diluted to 100 µl with water. 2.5 µl of diluted cDNA mixture was used for 25-µl PCR reaction. PCR primers are as follow: 5′-GGC TGA GAC AAC AGG ACA TCG-3′ (F) and 5′-GAC CGT CGC TCA TCT CGT AAAA-3′ (R). The cycling condition is 95°C 5 min, (95°C 30 s, 64°C 30 s, 72°C 40 s×3 cycles, (95°C 30 s, 61°C 30 s, 72°C 40 s)×3 cycles, (95°C 30 s, 58°C 30 s, 72°C 40 s)×3 cycles, (95°C 30 s, 55°C 30 s, 72°C 40 s)×24cycles, 72°C 7 min. GAPDH was used as an internal control. The primers sequence are as follow: 5′-GAC CAC AGT CCA TGC CAT CAC-3′ (F), and 5′-GTC CAC CAC CCT GTT GCT GTA-3′ (R). The cycling condition is 95°C 5 min, (95°C 30 s, 63°C 30 s, 72°C 40 s)×25cycles, 72°C 7 min.

### Bisulfite modification, methylation specific PCR (MSP) and bisulfite sequencing (BSSQ)

Genomic DNA was prepared by the proteinase-K method. Genomic DNA was bisulfite-modified as described previously. MSP primers were designed according to genomic sequences around transcriptional start sites (TSS) and synthesized (BGI, Beijing, China) to detect unmethylated (U) and methylated (M) alleles. MSP primers are as follow: 5′-GCG CGT GTA GAT TTC GTT TTT CGC-3′ (MF); 5′-AAC CCC ACG AAC GAC GCCG-3′ (MR); 5′-TTG GGG TGT GTG TAG ATT TTG TTT TTTGT-3 (UF) and 5′-CCC AAA CCC CAC AAA CAA CAC CA-3′ (UR). The size of unmethylation PCR product is 161 bp and methylation PCR product is 152 bp. Bisulfite-treated DNA was amplified using BSSQ primers flanking the targeted regions, including MSP primers sites and the transcription start site. Sequencing primers were as follows: 5′-GGG GGA GGT YGY GGT GAT TT-3′ (F) and 5′-ACC TAC RAC RAT CCC AAC CC-3′ (R). Bisulfite sequencing was performed as previously described [11].

### Immunohistochemistry (IHC)

Immunohistochemistry (IHC) was performed on 5 µm thick serial sections derived from formaldehyde-fixed paraffin blocks of papillary thyroid cancer and paired adjacent tissue. After deparaffinization and rehydration, endogenous peroxidase activity was blocked for 30 min in methanol containing 0.3% hydrogen peroxide. After antigen retrieval, a cooling-off period of 20 min preceded the incubation with the primary antibody. DACT2 antibody (OriGene Tech, MD, USA) was used at a 1/1000 dilution overnight at 4°C. The staining intensity and extent of the stained area were graded according to the NIS-ELEMENTS Imaging software (Nikon, Tokyo, Japan) staining intensity of the cytoplasm (weak staining = 1; moderate staining = 2; strong staining = 3); extent of stained cells (0% = 0, 0–5% = 1, 5–10% = 2, 10–15% = 3, 15–20% = 4). The final immuno-reactive score (0 to 12) was determined by multiplying intensity score to the extent of stained cells score.

### Construction of Lentiviral DACT2 Expression Vectors

Human full-length *DACT2* cDNA(GenBank accession number NM_214 462) was amplified from pCMV-DACT2 vector [11]. Primers were as follow: 5′-TGA TCA ATG TGG ACG CCG GGC-3′ (F) and 5′-GTC GAC TCA CAC CAT GGT CAT GAC-3′ (R). The PCR product was then subcloned into the pLenti6-GFP vector. The inserts were verified by restriction digestion and DNA sequencing.

### Lentiviral infections and stable expression cells selection

293T cell line was maintained in 90% DMEM (Invitrogen, Carlsbad, CA, USA) supplementing with 10% fetal bovine serum at 37°C with 5% CO2. DACT2 expressed Lentivirus vector was transfected into HEK293T cells (5×10^6^ per 100 mm dish) using Polyethyleneimine (P.E.I.) solution at a 3∶1 ratio (P.E.I. mass: DNA mass). After 48 h, viral supernatant was collected and filtered. Then viral supernatant was added to the growing medium of TPC-1 cells and stably expressed cells were selected by Blasticidin (Life Technologies, Gaithersburg, MD, USA) at 0.2 ug/ml for 2 weeks.

### Cell viability assay

Cell viability was measured daily for 72 hours using MTT (3-(4,5-dimethylthiazol-2-yl)-2,5-diphenyltetrazolium bromide) Kit (KeyGEN Biotech, Jiangsu, China) according to the manufacturer’s instruction. The results were plotted as means ± SD.

### Colony formation assay

DACT2 stably expressed TPC-1 cells and DACT2 unexpressed TPC-1 cells were diluted and reseeded at 200 cells per well in 6-well culture plates in triplicates. Growth medium, conditioned with Blasticidin at 0.2 ug/ml, was exchanged every 24 h. After 14 d, cells were fixed with 75% ethanol for 30 min and stained with 0.2% crystal violet for visualization and counting.

### Flow cytometry analysis

After 12 hours of synchronization by serum starvation, DACT2 stably expressed TPC-1 cells and DACT2 unexpressed TPC-1 cells were cultured with 10% fetal bovine serum (FBS) for 24 h. Cells were fixed with 70% ethanol and stained with 50 µg/ml propidium iodide (KeyGEN Biotech, Jiangsu, China). The cells were then sorted by FACS Caliber (BD Biosciences, San Jose, CA) and analyzed by Modfit software.

### Wound healing assay

TPC-1 cells were grown to confluent monolayers on 6-well plates and a pipette tip was used to create linear scratch wounds. Medium without fetal bovine serum was used to inhibit cell proliferation. Wound images were taken with a digital camera mounted on light microscope at 0, 36 and 72 hours. The wound gap widths were measured using Image J software.

### Transwell assay

Migration: TPC-1 cells were added to the upper chamber of 8.0 µm pore size Transwell apparatus (Corning, NY, USA) at a density of 4×10^4^ cells (100 ul) per chamber and incubated for 13 hours followed by removal of the cells that remained in the top chamber with cotton swabs. Invasion: The upper chamber was coated with Matrigel (BD Biosciences, San Jose, CA). TPC-1 cells were added to the upper chamber at a density of 6×10^4^ cells (100 ul) per chamber and incubated for 36 hours followed by removal of the cells that remained in the top chamber with cotton swabs. Cells that penetrated to the lower membrane surface were fixed in 4% paraformaldehyde, stained with 0.2% crystal violet, and counted in high powered fields (100×) with light microscope.

### Dual-Luciferase reporter assay

Tpc-1 cells were seeded at 8×10^4^ cells per well in 24-well culture plates 24 h before transfection. To examine transcriptional activity driven by TCF/LEF, Tpc-1 cells were transfected with 100 ng/well TOP flash reporter vector (TCF/LEF–responsive reporter), 10 ng/well pRL-TK and pCI-neo-β-catenin expressing vector (150 ng/well) was used to activate the reporter gene; As a negative control, another group of Tpc-1 cells were transfected with 100 ng/well Fopflash reporter vector, 10 ng/well pRL-TK control vector and β-catenin expressing vector (150 ng/well). Then, increasing amounts of 150 ng/well DACT2 vector and 150 ng/well Dvl2 vector were transfected into cells together with Topflash reporter vector, pRL-TK control vector and pCI-neo-β-catenin to evaluate the regulative function of DACT2 in Wnt signaling pathway.

48 h after transfection, relative luciferase activities were measured with the Dual Luciferase Reporter Assay system (Promega, Shanghai, China) according to the manufacturer’s protocol. For each experiment, the luciferase reporter assay was performed three times [35].

### DACT2 knocked down by siRNA

One selected siRNA (siRNA618) [11] targeting DACT2 and RNAi Negative Control Duplex were used in this study. The sequences were as follows: siRNA duplex (sense: 5-GUC GGU UGA UGA GAC UAC UTT-3; antisense: 5-AGU AGU CUC AUC AAC CGA CTT-3); RNAi negative control duplex (sense: 5-UUC UCC GAA CGU GUC ACG UTT-3; antisense: 5-ACG UGA CAC GUU CGG AGA ATT-3). RNAi oligonucleotide or RNAi negative control duplex (Gene Pharma Co, Shanghai, China) was transfected into W3 cells according to the manufacturer’s instructions.

### Protein preparation and western blotting

Transfected cells were lysed in RIPA Lysis Buffer (Beyotime Biotech, Jiangsu, China). The protein lysates were then separated by SDS-PAGE and electro-blotted onto PVDF membranes. After blocking with 5% nonfat milk and 0.1% Tween-20 in TBS, the membranes were incubated with antibodies. The antibodies were as follows: DACT2 (OriGene Tech, MD,USA), cyclinD1 (Bioworld Tech, MN, USA), c-myc (Bioworld Tech, MN, USA), MMP-9 (Bioworld Tech, MN, USA), β-catenin (Bioworld Tech, MN, USA), phospho-β-catenin (Bioworld Tech, MN, USA) and β-actin (Beyotime Biotech, Jiangsu, China).The blots were visualized using enhanced chemiluminescence (Beyotime Biotech, Jiangsu, China).

### Statistical Analysis

The association of DNA methylation and clinic-pathologic factors in human papillary thyroid cancer was analyzed by the *χ^2^* test for independence dichotomous variables and the Student’s t test for continuous variables. Results were judged to be statistically significant at p<0.05(*), p<0.01(**), p<0.001(***). All analyses were carried out using SPSS 19.0 software.

## Results

### *DACT2* is silenced by promoter region hypermethylation in thyroid cancer cells

**Figure 1:**
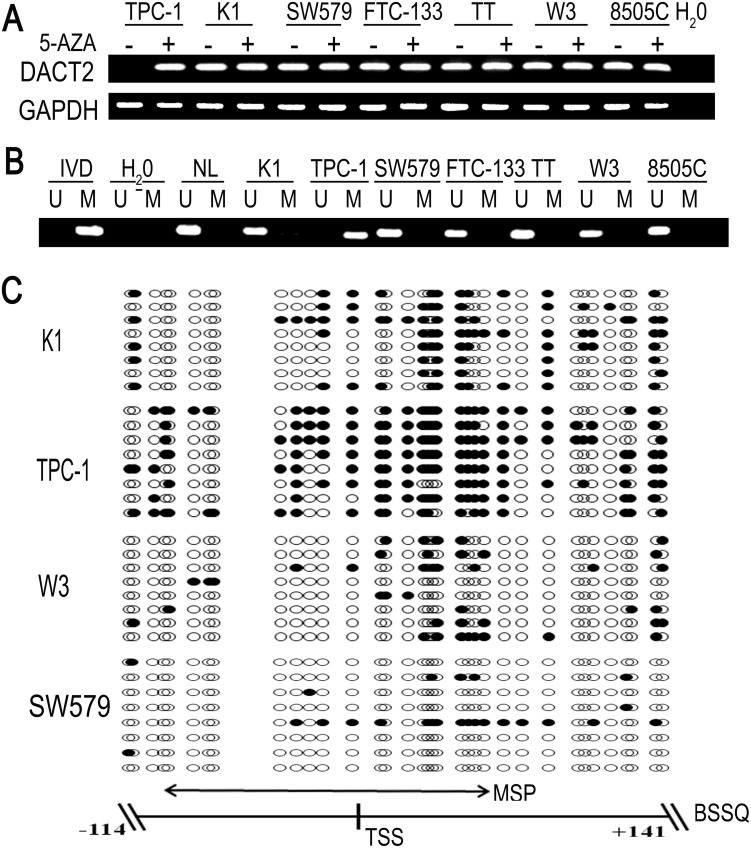
*DACT2* expression and promoter region methylation status in thyroid cancer cell lines. **A:** *DACT2* expression was analyzed by regular PCR before(−) and after (+) 5-Aza treatment. K1,TPC-1,SW579,FTC-133,TT,W3 and 8505C are thyroid cancer cell lines. H2O: double-distilled water. GAPDH: internal control. **B:** MSP results show *DACT2* methylation status. IVD (invitro methylated DNA) served as methylation control. NL (normal lymphocyte DNA) served as unmethylation control. M: methylated alleles; U: unmethylated alleles. **C:** Representative bisulfite sequencing results in *DACT2* promoter region. Double-headed arrow represents MSP amplification region. Filled circles represent methylated CpG sites and open circles denote unmethylated CpG sites. TSS: transcriptional start site.

To explore the expression of *DACT2* in thyroid cancer, regular PCR was employed. *DACT2* expression was discovered in the human thyroid cancer cell line K1, SW579, FTC-133, TT, W3 and 8505C by regular PCR. Loss of *DACT2* expression was detected in the TPC-1 cell line (Fig. 1A). We next examined *DACT2* methylation using Methylation-Specific PCR (MSP) in these cell lines. Complete methylation was found in TPC-1 cells and unmethylation was found in K1, SW579, FTC-133, TT, W3 and 8505C cell lines (Fig. 1B). Loss of *DACT2* expression was correlated with promoter region hypermethylation. To further validate *DACT2* expression was regulated by promoter region methylation, thyroid cancer cell lines were treated by 5-aza-2′-deoxycytidine (5-Aza). Re-expression of *DACT2* was found in TPC-1 cell line and no expression changes were revealed in K1, SW579, FTC-133, TT, W3 and 8505C cell lines after 5-Aza treatment (Fig. 1A). These results indicate that *DACT2* expression was regulated by promoter region methylation. To see the efficiency of methylation specific PCR (MSP) primers and the methylation density in *DACT2* promoter region, we performed bisulfite sequencing (BSSQ; Fig. 1C). MSP results are consistent with bisulfite sequencing results very well and *DACT2* promoter region was densely methylated in TPC-1 cells. Spare methylation sites in the promoter region were found in K1, W3 and SW579 cells.

### *DACT2* is frequently methylated in primary papillary thyroid cancer and methylation of DACT2 is associated with lymph node metastasis

**Figure 2:**
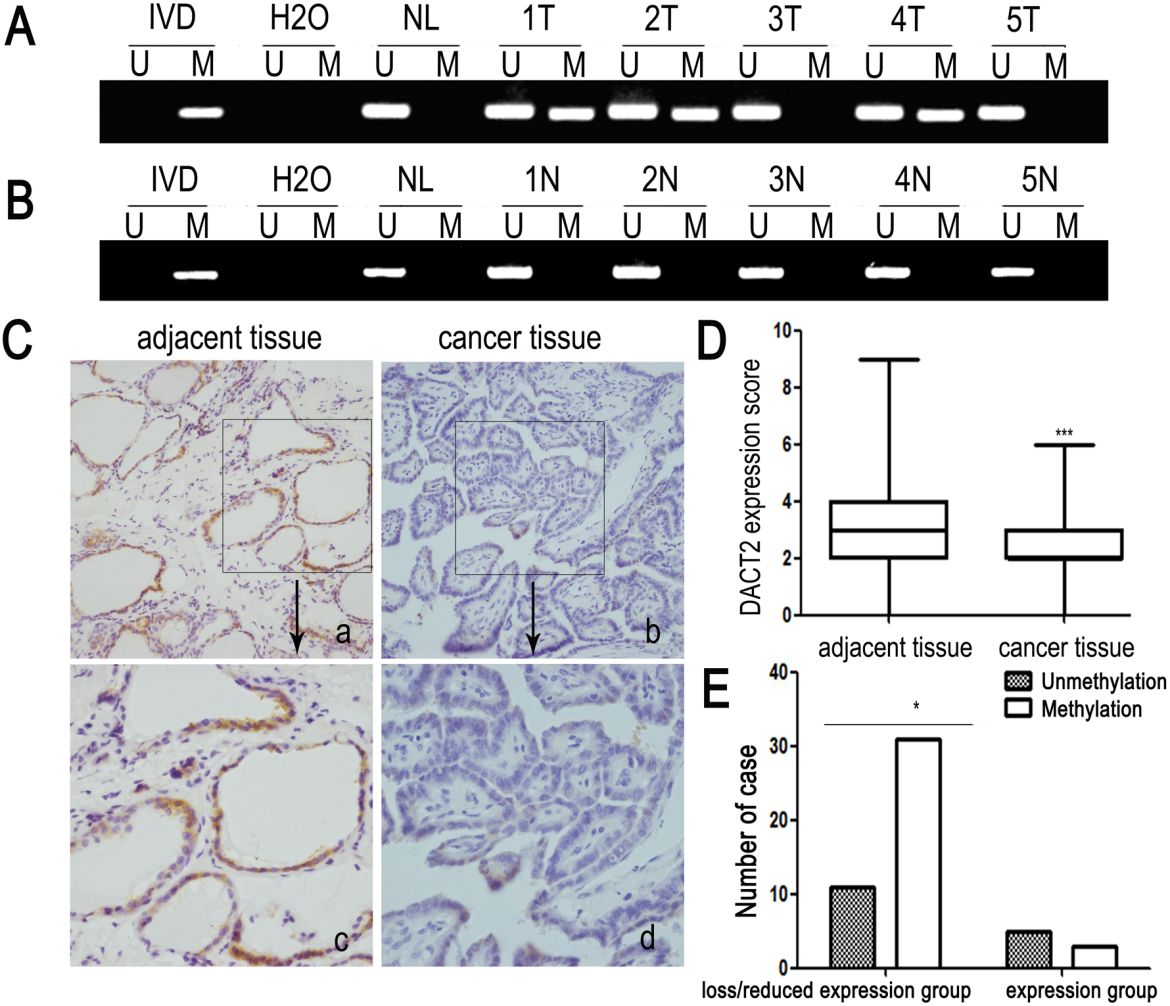
Representative MSP and IHC results in primary papillary thyroid cancer. **A:** Representative MSP results of *DACT2* in human primary papillary thyroid cancer. **B:** Representative MSP results of *DACT2* in normal thyroid tissues from noncancerous patients. **C:** Representative DACT2 staining results in papillary thyroid cancer and adjacent tissues determined by IHC. (a, b, 200×; c, d, 400×). **D:** DACT2 expression scores are shown as box plots, horizontal lines represent the median score; the bottom and top of the boxes representing the 25th and 75th percentiles, respectively; vertical bars represent the range of expression. DACT2 expression is significantly different in 50 cases of matched tumor and adjacent tissue samples, p<0.001. **E:** The association of loss/reduction of DACT2 expression and promoter hypermethylation was analyzed *x^2^* test in 50 cases of matched primary thyroid papillary cancer and adjacent tissue samples. (p<0.05).

To explore methylation changes in *DACT2* in thyroid cancer development, 99 cases of primary papillary thyroid cancer were detected by MSP. 64 cases of advanced thyroid cancer (64.6%) were methylated (Fig. 2A). No methylation was found in 10 cases of non-cancerous thyroid tissue samples (Fig. 2B). As shown in Table 1, methylation of *DACT2* was associated with lymph node metastasis significantly (p<0.01). No association was found between *DACT2* methylation and age, gender, smoking, drinking, family history and radiation exposure history. The results suggest that methylation of *DACT2* is associated with thyroid cancer metastasis. Above results indicate that methylation of *DACT2* may serve as detective and metastatic marker of papillary thyroid cancer.

**Table 1:** The association of DACT2 methylation and clinical factors in 99 cases of papillary thyroid cancer.

		*DACT2* methylation status		
Clinical Factor	No.	methylated	unmethylated	p value
		n = 64(64.6%)	n = 35(35.4%)	(*χ^2^* test)
**Age(years)**				
<46	56	39	17	0.235
≥46	43	25	18	
**Gender**				
Male	28	17	11	0.607
Female	71	47	24	
**Tumor Size(cm)**				
≤1	33	21	12	0.882
>1	66	43	23	
**Lymph Node Metastasis**				
Negative	50	25	25	0.002**
Positive	49	39	10	

*DACT2* methylation is associated with lymph node metastasis of papillary thyroid cancer (p = 0.002).

**p values are obtained from *x^2^* test, significant difference, p<0.01.

In order to explore the association of DACT2 expression and promoter region hypermethylation in primary papillary thyroid cancer, DACT2 expression was evaluated by immunohistochemistry (IHC) in 50 cases of available matched papillary thyroid cancer and adjacent tissue samples. DACT2 staining was observed predominantly in the cytoplasm as reported in the other cancers. We found that DACT2 expression was reduced in papillary thyroid cancer tissues compared with the adjacent tissue samples (p<0.001, Fig. 2C and D). And reduced expression was associated with DACT2 promoter region hypermethylation significantly (p<0.05; Fig. 2E). These results suggest that DACT2 expression is regulated by promoter region hypermethylation in primary papillary thyroid cancer.

### Restoration of DACT2 expression suppresses cell growth and induces G1 arrest in thyroid cancer cells

**Figure 3:**
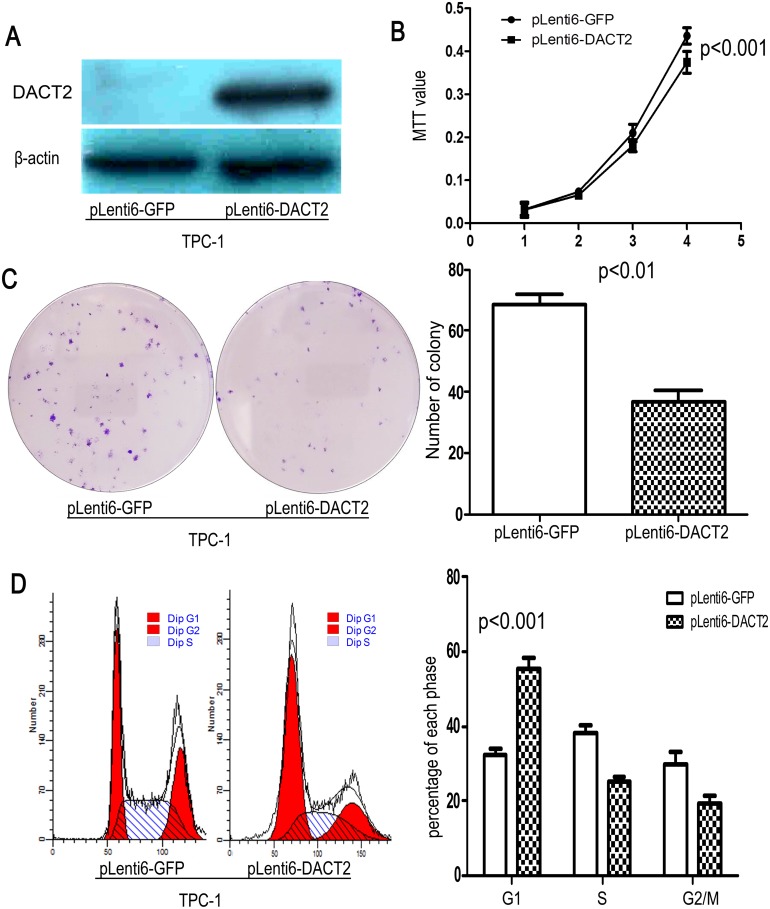
DACT2 suppresses thyroid cancer cell proliferation. **A:** Re-expression of DACT2 was found after transfection of DACT2 expression vector in TPC-1 cells. **B:** MTT results: DACT2 suppresses the viability of TPC-1 cells (p<0.001). **C:** Colony formation results before and after re-expression DACT2 in TPC-1 cells. Each experiment was repeated for three times (p<0.01). White bar: empty vector; black bar: DACT2 expression vector. **D:** G1 arrest was induced by DACT2 in TPC-1 cells. Each experiment was repeated for three times (p<0.001). White bar: empty vector; black bar: DACT2 expression vector.

To evaluate the effect of DACT2 on thyroid carcinogenesis, cell viability and colony formation were evaluated before and after restoration of DACT2 expression in TPC-1 cells (Fig. 3A). Cell viability and colony formation was suppressed after re-expression of DACT2 in TPC-1 cells (Fig. 3B and C, p<0.01). The results indicate that DACT2 inhibits thyroid cancer cell proliferation. To further understand the mechanism, flow cytometry assay was employed. The distribution of cell phases in DACT2 unexpressed and re-expressed TPC-1cells were as follows: G0/1 phase: 32.24±1.61% *vs.* 55.49±3.09% (p<0.001); S phase: 38.06±2.34% *vs.* 25.23±1.15% (p<0.05); G2/M phase: 29.70±3.38% *vs*. 19.27±2.32% (p<0.05). The results suggest that G0/1 phase was increased, S phase and G2/M phase were reduced after restoration of DACT2 expression in TPC-1cells (Fig. 3D). As shown in Figure S1, no apoptosis was induced by DACT2 in TPC-1 cells (p>0.05).

### Restoration of DACT2 expression inhibits cell migration and invasion in papillary thyroid cancer

**Figure 4:**
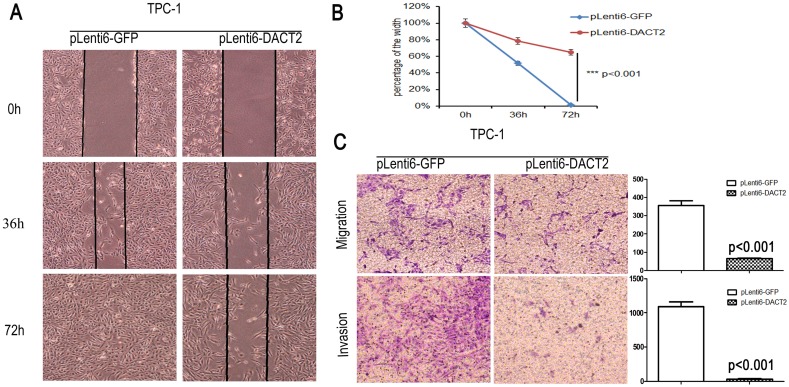
Cell migration and invasion were suppressed by DACT2 in TPC-1 cells. **A:** Cell migration was suppressed by DACT2 significantly in TPC-1 cells under the wound healing detection. **B:** Blue line represents the wound healing result of empty vector group and the red line represents the wound healing result of DACT2 re-expressed group in TPC-1 cells for 72 hours (p<0.001). **C:** Cell migration and invasion was suppressed by DACT2 in TPC-1 cells under the transwell study (p<0.001).

As DACT2 methylation is associated with papillary thyroid cancer metastasis, the effect of DACT2 on cell invasion and migration was analyzed in papillary thyroid cancer cells. Wound healing and transwell assay were employed in this study. As shown in Fig. 4A and 4B, the cell migration was inhibited apparently after re-expression of DACT2 in TPC-1 cells (p<0.001). In the transwell assay without ECM coating, the number of migrated cells of each high powered field was 354.50±47.44 *vs.* 64.83±4.48 before and after re-expression of DACT2 in TPC-1 cells (Fig. 4C, p<0.001). Under the invasion detection, the invasive cell number of each high powered field was 1091.40±119.55 *vs.* 31.58±16.36 before and after restoration of DACT2 expression (Fig. 4C, p<0.001). It indicates that DACT2 inhibits cell invasion and migration in thyroid cancer. Above results hint that DACT2 suppresses thyroid cancer metastasis.

### DACT2 inhibits Wnt/β-catenin signaling in thyroid cancer

**Figure 5:**
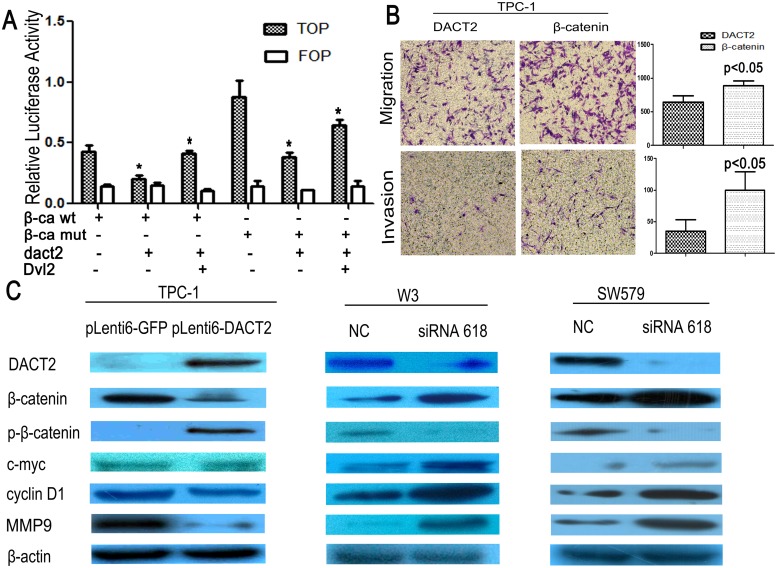
DACT2 inhibit Cell migration and invasion by inhibiting Wnt pathway in TPC-1 cells. **A:** Results of TCF/LEF luciferase reporter assay. β-catenin expression vector was co-transfected with TCF/LEF Topflash reporter or its mutant, Fopflash in TPC-1 cells. Luciferase activity was normalized to Renilla luciferase activity. Relative luciferase activity (the ratio of firefly luciferase to renilla luciferase) was suppressed by DACT2. The experiment was repeated for three times (*p<0.05). Increased luciferase activity was induced by co-transfecting DACT2 and Dvl2. The experiment was repeated for three times (*p<0.05). **B:** Cell migration and invasion was increased by β-catenin in DACT2 stably expressed TPC-1 cells under the transwell study. The experiment was repeated for three times (p<0.05). **C:** The expression of β-catenin, c-myc, cyclinD1 and MMP-9 were decreased and the level of phosphorylated β-catenin(p-β-catenin) was increased after the restoration of DACT2 expression in TPC-1 cells. The expression of β-catenin, c-myc, cyclinD1 and MMP-9 were increased and the level of p-β-catenin was reduced after knock down DACT2 in W3 and SW579 cells.

In the canonical Wnt/β-catenin signaling pathway, increased β-catenin in the cytoplasm will promote translocation of β-catenin into the nucleus. β-catenin in nuclei binds to the TCF/LEF in several types of cancers for transcriptional activation of downstream genes, such as cyclinD1, c-myc and MMPs [15]–[21], which play important roles in carcinogenesis and metastasis. To explore the effect of DACT2 on Wnt signaling in human thyroid cancer, Topflash and (TCF/LEF) reporter system was employed. As shown in Figure 5A, the activity of TCF/LEF was significantly inhibited by DACT2. It is similar with our previous report in human lung cancer [11], the activity of TCF/LEF was increased by co-transfecting DACT2 and Dvl2 with wild-type or mutant-type β-catenin. To further validate the effect of DACT2 on cell migration and invasion, β-catenin was overexpressed in DACT2 stably expressed TPC-1 cells. The number of migrated cells of each high powered field was 646±158 *vs.* 867±118 before and after overexpression of β-catenin in DACT2 stably expressed TPC-1 cells (Fig. 5B, p<0.05). The number of invasive cell for each high powered field was 35±32 *vs.* 100±50 before and after overexpression of β-catenin in DACT2 stably expressed TPC-1 cells (Fig. 5B, p<0.05). The results further suggest that DACT2 suppresses cell migration and invasion by inhibiting Wnt signaling in human thyroid cancer. Phosphorylated β-catenin (p-β-catenin) is a major component representing β-catenin degradation. In our study, the expression of c-myc, cyclinD1 and MMP-9 was reduced, and the level of p-β-catenin was increased after re-expression of DACT2 in TPC-1 cells (Fig. 5C). To further validate the effect of DACT2 on Wnt signaling, siRNA knockdown technique was employed. The expression of c-myc, cyclinD1 and MMP-9 was increased, and the level of p-β-catenin was reduced in DACT2 expressed W3 and SW579 cells (Fig. 5C). Above results suggest that DACT2 suppresses thyroid cancer cell proliferation and metastasis by inhibiting Wnt signaling.

## Discussion

It is desirable to manage thyroid cancer personally based on biomarkers. So far no reliable biomarker was found for predicting prognosis and radio or chemo-sensitivity in thyroid cancer [22], [23]. Epigenetic changes were found frequently in human cancers, including thyroid cancer [24]–[29]. DNA methylation may serve as diagnostic, prognostic, chemo-sensitive and radio-sensitive markers [30]–[35]. Silenced expression of the key component by DNA methylation in cancer related signaling pathway may become the therapeutic target [34]–[37].

Xenopus Dapper and Xenopus Frodo are Dishevelled-binding proteins, showing 89% total-amino-acid identity [38]. Xenopus Dapper is claimed to inhibit the β-catenin pathway as well as the JNK pathway, while Xenopus Frodo is claimed to activate the β-catenin pathway [6], [39]. DACT1 and DACT2 are human homologs of Xenopus Dapper and Frodo, which are Dishevelled-binding proteins. DACT1 and DACT2 genes, consisting of four exons, were found to encode DACT1 protein (799-amino-acids) and DACT2 protein (774-amino-acids), respectively. DACT1 and DACT2 showed 28.8% total-amino-acid identity. Seven DAPH domains, including DAPH2 (leucine zipper), DAPH3 (serine rich) and DAPH7 (PDZ binding), were conserved between DACT1 and DACT2. DACT1 and DACT2 genes were mapped to human chromosome 14q22.3 and human chromosome 6q27, respectively [7]. Human chromosome 14q22.3–32.1 is deleted in astrocytoma [40]. Human chromosome 6q27 is a region of frequent loss of heterozygosity in human cancers [41]–[47]. Based on Evolutionary and functional conservation of Wnt signaling molecules as well as human chromosomal localization, DACT1 and DACT2 genes were predicted to be potent cancer-associated genes. Recent study suggested that DACT2 inhibits Pitx2 activating Wnt signaling in embryonic tooth development [8]. Our previous studies found that DACT2 is frequently methylated in lung and hepatic cancer, and methylation of DACT2 activates Wnt signaling [10], [11]. To further understand the role of DACT2 in thyroid cancer, we detected the promoter region methylation in papillary thyroid cancer first. DACT2 is frequently methylated in human papillary thyroid cancer and methylation of DACT2 is associated with lymph node metastasis. These findings suggest that DACT2 methylation may serve as detective and prognostic marker of papillary thyroid cancer. The wound healing and transwell assay were employed to evaluate the effect of DACT2 on thyroid cancer metastasis. As expected, re-expression of DACT2 inhibits cell migration and invasion in TPC-1cells. The expression of MMP-9 was reduced after restoration of DACT2 expression in TPC-1cells and increased after knockdown of DACT2 in W3 and SW579 cells. As DACT2 expression was regulated by promoter region hypermethylation in papillary thyroid cancer, DACT2 methylation may involve in thyroid carcinogenesis. To answer these questions, the function of DACT2 was further studied. Cell proliferation and colony formation was inhibited, and G1 phase arrest was induced by DACT2 in thyroid cancer cells. These results demonstrate that DACT2 is a tumor suppressor in thyroid cancer. To further understand the mechanism of DACT2 on thyroid carcinogenesis, the effect of DACT2 on the Wnt signaling was analyzed. The activity of TCF/LEF reporter was inhibited by DACT2 and increased after overexpression of Dvl2 according to the Topflash reporter system analysis in TPC-1 cells. The expression of β-catenin and its downstream targets, c-myc, cyclinD1 and MMP-9, were suppressed by DACT2 in TPC-1cells. The role of DACT2 in Wnt signaling was further validated by siRNA knocking down in W3 and SW579 cells. Overexpression of β-catenin promotes cell migration and invasion in DACT2 stably expressed TPC-1 cells. Above results suggest that DACT2 inhibits thyroid carcinogenesis and metastasis by inhibiting Wnt signaling pathway. It is possible to treat or prevent papillary thyroid cancer by targeting de-methylation of DACT2 to inhibit Wnt signaling.

In conclusion, DACT2 is frequently methylated in human papillary thyroid cancer and methylation of DACT2 is related to lymphoid node metastasis. DACT2 expression was regulated by promoter region methylation. DACT2 suppresses cell proliferation and metastasis by inhibiting Wnt signaling in thyroid papillary cancer.

## Supporting Information

Figure S1 **The effect of DACT2 on apoptosis in TPC-1 cells.** Flow cytometry assay shows: no significant difference was found in cell apoptosis in DACT2 expressed and unexpressed TPC-1 cells (p>0.05), this experiment was repeated for three times.
(TIF)
