# Supplementary figures and images for: Methylation of DACT2 Promotes Papillary Thyroid Cancer Metastasis by Activating Wnt Signaling
Source: PLoS One. 2014 Nov 6;9(11):e112336. doi: 10.1371/journal.pone.0112336 (PMC4223043; doi:10.1371/journal.pone.0112336)

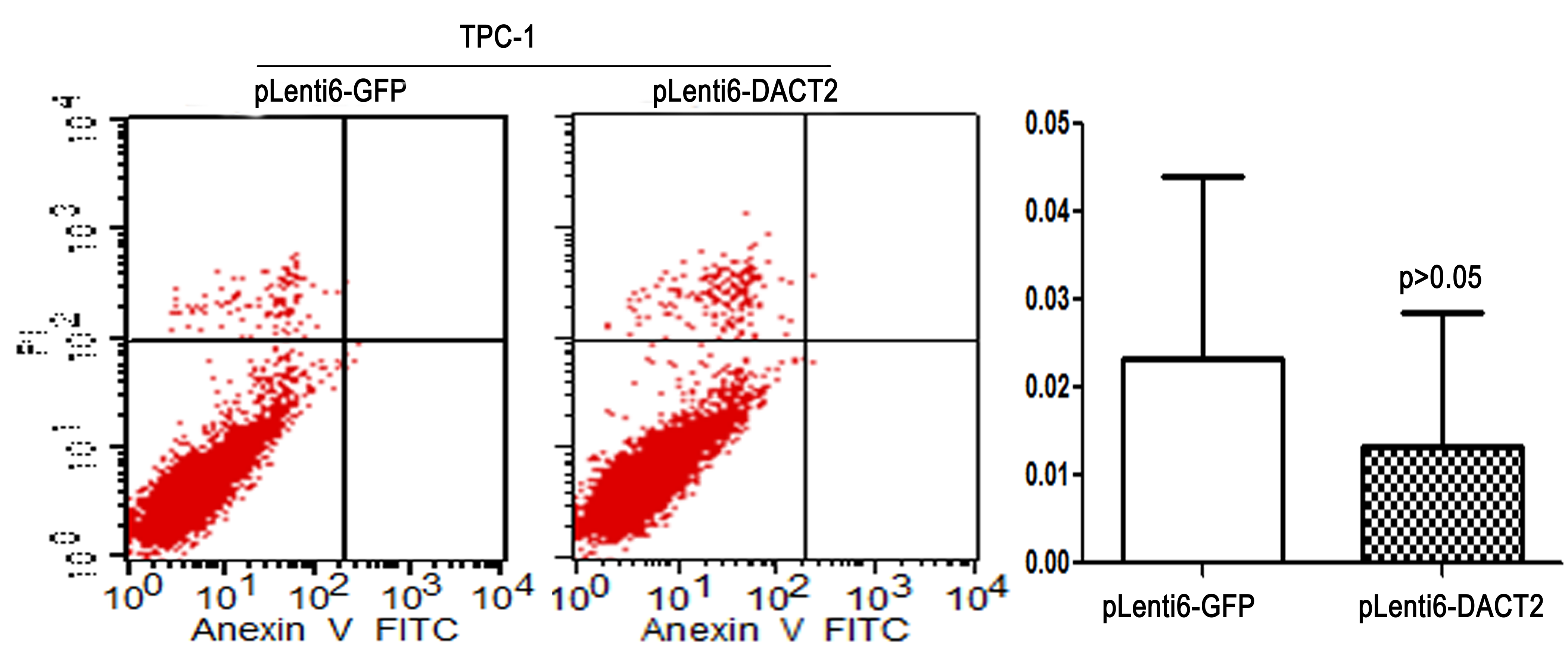

Supplement: Figure S1 — The effect of DACT2 on apoptosis in TPC-1 cells. Flow cytometry assay shows: no significant difference was found in cell apoptosis in DACT2 expressed and unexpressed TPC-1 cells (p>0.05), this experiment was repeated for three times. (TIF) [file pone.0112336.s001.tif]
